# Role of RHO family interacting cell polarization regulators (RIPORs) in health and disease: Recent advances and prospects

**DOI:** 10.7150/ijbs.65457

**Published:** 2022-01-01

**Authors:** Zeheng Lv, Yan Ding, Wenxin Cao, Shuyun Wang, Kun Gao

**Affiliations:** 1 Department of Clinical Laboratory, Shanghai First Maternity and Infant Hospital, School of Medicine, Tongji University, Shanghai 200092, China.; 2 Department of Breast Surgery, Shanghai First Maternity and Infant Hospital, School of Medicine, Tongji University, Shanghai 200092, China.

**Keywords:** RIPOR1, RIPOR2, RIPOR3, RHO GTPase, RHOA, RHOC, 14-3-3 protein, polarization, migration

## Abstract

The RHO GTPase family has been suggested to play critical roles in cell growth, migration, and polarization. Regulators and effectors of RHO GTPases have been extensively explored in recent years. However, little attention has been given to RHO family interacting cell polarization regulators (RIPORs), a recently discovered protein family of RHO regulators. RIPOR proteins, namely, RIPOR1-3, bind directly to RHO proteins (A, B and C) via a RHO-binding motif and exert suppressive effects on RHO activity, thereby negatively influencing RHO-regulated cellular functions. In addition, RIPORs are phosphorylated by upstream protein kinases under chemokine stimulation, and this phosphorylation affects not only their subcellular localization but also their interaction with RHO proteins, altering the activation of RHO downstream targets and ultimately impacting cell polarity and migration. In this review, we provide an overview of recent studies on the function of RIPOR proteins in regulating RHO-dependent directional movement in immune responses and other pathophysiological functions.

## Introduction

Cell migration plays a fundamental role in both normal physiological processes such as immune responses and abnormal pathologies such as tumor metastasis 1. Establishing and maintaining front-rear polarity is integral to the movement of cells in specific directions. Cell polarization necessitates dynamic reorganization of the actin cytoskeleton, temporal changes in gene transcription, spatial localization of specific proteins, and reorientation of secretory trafficking 2.

The mechanisms underlying cell polarization and migration involve cooperation among multiple proteins, such as the small GTP-binding proteins RHO GTPases 3. In mammals, 20 members of the RHO GTPase family have been identified and classified into eight subfamilies: RHO, RHOBTB, RHODF, RND, RHOUV, RHOH, CDC42 and RAC 3,4. A better understanding of RHO GTPase function and how their activities are regulated is needed to elucidate the mechanisms of cell polarization and migration.

RHO GTPases act as molecular switches to control multiple cellular events by switching between an inactive GDP-bound state and an active GTP-bound state 5-7. This cycling activity is strictly regulated by three groups of factors: guanine nucleotide exchange factors (GEFs), which promote GDP/GTP exchange; GTPase-activating proteins (GAPs), which facilitate the hydrolysis of GTP to GDP; and guanine nucleotide dissociation inhibitors (GDIs), which prevent nucleotide exchange and regulate relocalization of RHO GTPases, thereby inhibiting their access to downstream effectors.

One of the most well-characterized subfamilies of RHO GTPases is the RHO subfamily, which contains three highly homologous proteins: RHOA, RHOB, and RHOC. In addition to other classic RHO regulators, recent studies have linked RHO family interacting cell polarization regulators (RIPORs) as novel partners of RHO subfamily members 8-11. The RIPOR family is composed of RIPOR1 (also called FAM65A), RIPOR2 (also called FAM65B, C6orf32, KIAA0386, or PL48), and RIPOR3 (also called FAM65C).

In this review, we summarize recent research on the functions of RIPOR1 and RIPOR2 in the RHO signaling pathway, with a focus on advances in deciphering the mechanisms of RIPOR proteins in RHO-dependent directional movement in immune responses, tumor cell metastasis and other pathophysiological functions.

## The RIPOR family, a novel family of RHO-binding proteins

### History of discovery

In 1997, RIPOR2 was first cloned from differentiating cytotrophoblasts and reported as a novel gene with markedly elevated mRNA expression during the differentiation of DMSO-induced HL-60 cells 12. RIPOR2 was then found to induce the formation of neurite-like protrusions in the murine myoblast cell line C2C12 in 2007 and in quail myoblast QM-RSV cells in 2008 13,14; however, little is known about how RIPOR2 modulates cell morphogenesis. Later studies by Rougerie et al. and our research group demonstrated that RIPOR2 directly bound to RHOA in neutrophils 10. RIPOR2 led to a reduction in active RHOA and RHOA-mediated transcriptional activation, suggesting that RIPOR2 is a RHOA inhibitor. Moreover, inhibition of RHOA activity, as well as overexpression of RIPOR2, suppressed T cell migration, further confirming the involvement of RIPOR2-RHOA in cell polarization 8. Collectively, these findings demonstrated that RIPOR2 is involved in regulating T cell and neutrophil migration through RHOA signaling. Since these findings, an increasing number of studies have been carried out to decipher the important roles of RIPORs in cell migration and polarization. In 2016, Mardakheh et al. revealed that RIPOR1 directly interacted with active RHOA, RHOB and RHOC 11. In contrast, the functions of RIPOR3 have not yet been elucidated.

### Structural basis for the regulation of the RHO subfamily by RIPOR proteins

**Figure 1:**
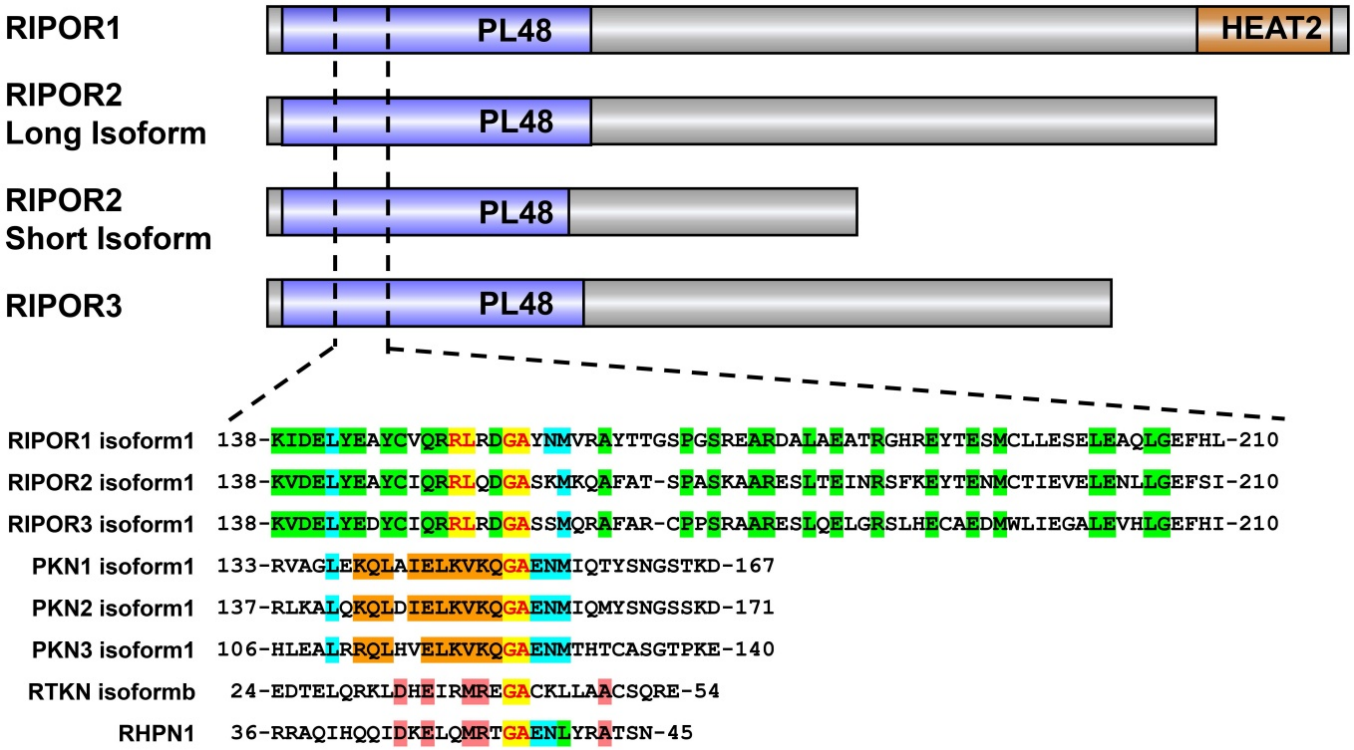
The domain architecture of RIPOR family and sequence alignment of amino acids of the RBM in RIPORs (RIPOR1: NP_078795.2,RIPOR2: NP_055537.2,RIPOR3: NP_543019.2) with PKN1 (NP_998725.1), PKN2 (NP_006247.1), PKN3 (NP_037487.2) and the HR of RTKN (NP_149035.1) and RHPN1 (NP_443156.2).

RIPOR1, RIPOR2 and RIPOR3 share an identical RHO-binding motif in the N-terminal region (Figure 1), which shows moderate similarity to the RHOA-binding homology regions (HRs) in several known RHOA effectors, such as protein kinase N-1, -2, and -3 (PKN1, PKN2, PKN3), Rhotekin (RTKN1), and Rhophilin (RHPN1). The invariant glycine (Gly155 in RIPOR2) and alanine (Ala156 in RIPOR2) are highly conserved among these RHOA effectors. Except for these two amino acids, the amino acids within the RHO-binding motif (RBM, aa 138-210) in RIPORs are also highly homologous, especially Arg151 and Leu152. Mutation of Arg151 and Leu152 to Ala or of Gly155 and Ala156 to Arg in RIPOR2 abolish the interaction between RIPOR2 and RHOA 10. The N-terminal parts of RIPORs are evolutionarily conserved, and more than 70% identity is observed among the RIPORs. In addition, a RIPOR1 mutant lacking the N-terminus failed to interact with RHOA and was unable to inhibit RHOA activity 10. These observations highlighted the importance of the N-terminal segment of RIPOR proteins. RIPOR3 shows high homology with RIPOR1/2 in the RBM, implying that RIPOR3 is a potential interacting protein of RHOA. Indeed, we confirmed this interaction between RIPOR3-RHO proteins through the RBM by coimmunoprecipitation assays (data not published). Finally, a brief phylogenetic analysis of RIPORs suggested that RIPORs are encoded only in vertebrate genomes and the RBM is highly conserved in all RIPORs irrespective of species (data not shown).

Analysis of the protein sequences of RIPORs also revealed some unique structures among RIPORs. For example, RIPOR1 contains a HEAT repeat domain in its C-terminal region, although the function of this domain has not been elucidated (Figure 1).

### Posttranslational modifications (PTMs) of RIPOR2

Based on PhosphoSitePlus data, the three RIPORs are potentially phosphorylated. Upon chemoattractant stimulation, RIPOR2 showed a motility shift and was stabilized in neutrophils. This phenomenon was phenocopied in cells treated with the phosphatase inhibitor okadaic acid 10. However, deletion of phospholipase C (PLC) β2/β3 and phosphatidylinositol-3-kinase (PI3K)γ impaired the motility shift of RIPOR2 10. Therefore, chemokines induce RIPOR2 phosphorylation in part through PKC and AKT 9-10,15. Treatment with the proteasome inhibitor MG132 stabilized RIPOR2, implying that its turnover is controlled by the proteasomal pathway 9-10. These findings shed light on the underlying mechanisms by which RIPOR2 phosphorylation might impede its degradation by the proteasome. Moreover, in cardiomyocytes, RIPOR2 can be phosphorylated by phosphatase and tensin homolog-induced putative kinase 1 (PINK1) to participate in autophagy 15.

Notably, stimulation with the chemokine CCL19 was found to disrupt the RIPOR2-RHOA interaction, an effect that was abrogated by phosphatase inhibitor treatment 9, implying that chemokine-induced RIPOR2 phosphorylation decreases the interaction between RIPOR2 and RHOA.

RIPOR2 phosphorylation also alters its subcellular localization during cell migration. Stimulation with the chemokine fMLP induced the accumulation of RIPOR2 at the leading edge of neutrophils. However, the distribution of the nonphosphorylated RIPOR2 S5A mutant (incapable of phosphorylation by PKC/AKT) was not affected by chemokine stimulation 10. Interestingly, phosphorylation was found to result in relocalization of RIPOR2 from the plasma membrane to the cytosol, thereby disrupting the interaction of RIPOR2 with RHOA 9. In summary, phosphorylation of RIPOR2 not only disrupts the RIPOR2-RHOA interaction but also impacts the subcellular localization of RIPOR2.

Data from the PhosphoSitePlus database indicate that the RIPOR1 and RIPOR3 proteins contain several phosphorylation sites 16. For this reason, we speculate that both RIPOR1 and RIPOR3 may be phosphorylated, although additional experiments are warranted.

Other PTMs, including ubiquitination of RIPOR1/2, acetylation of RIPOR2/3 and arginine methylation of RIPOR3, have also been deduced from high-throughput mass spectrometry data 16. However, the precise cellular consequence of these PTMs on RIPORs remains elusive.

### 14-3-3 family members are RIPOR-binding partners

**Figure 2:**
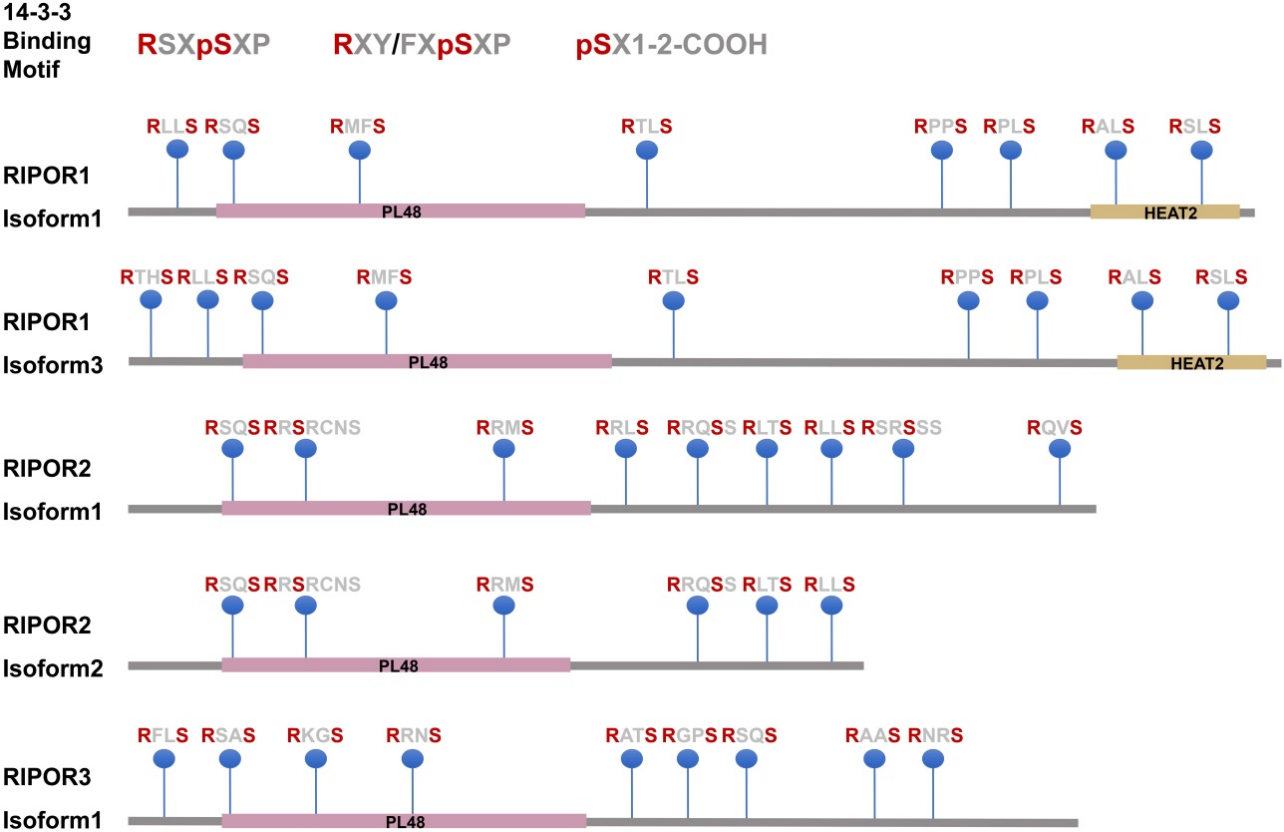
The potential 14-3-3 binding motifs in RIPORs.

14-3-3 family proteins (also called tyrosine 3- monooxygenase/tryptophan 5-monooxygenase activation proteins (YWHAs)) are highly conserved proteins that are widely expressed in eukaryotic cells 17,18. 14-3-3 family members were recently identified as novel interacting proteins of RIPOR1/2 10,11. These proteins bind to Ser/Thr-phosphorylated proteins as hetero- or homodimers by recognizing three types of conserved motifs: RSXpSXP, RXY/FXpSXP, and pSX1-2-COOH 17,19. Protein sequence analysis showed that RIPORs have many potential 14-3-3 binding sites (Figure 2). The 14-3-3 consensus sequences in RIPORs also match the substrate motifs for many protein kinases. Our work showed that RIPOR2 can be phosphorylated by PKC and AKT at serine residues 21, 37, 341, 523, and 535 in regions that match the 14-3-3 consensus sequences. Not surprisingly, the RIPOR2 S5A mutant showed a much lower affinity for 14-3-3 proteins 10. As regulatory molecules, 14-3-3 proteins modulate the conformation, activity, and subcellular localization of their interacting partners 20. Indeed, overexpression of 14-3-3 proteins led to higher levels of RIPOR2, implying that 14-3-3 proteins promote RIPOR2 stabilization 10. In addition, several 14-3-3 proteins interacted with RIPOR1 in a RHO-dependent manner 11, although the effects of 14-3-3 proteins on RIPOR1 have not been examined.

## The pathophysiological functions of RIPORs

### RIPOR2 and immune cell function

The biological significance of the role of RHO in T cell development, activation, and migration is well documented 21-23. RIPOR2 functions in the adhesion, polarization, and migration of T cells in a RHOA-dependent manner. Depletion of RIPOR2 by small interfering RNAs increased the percentage of cells exhibiting arrest *in vitro*. A similar effect was further verified in lymphocyte arrest on blood vessel endothelial cells 8. Inhibition of RHOA activity, as well as overexpression of RIPOR2, was found to impede T cell migration. In contrast, the RIPOR2 Δ113 mutant, which was unable to bind RHOA, did not exert this effect 8. RIPOR2 was found to inhibit GTP loading on RHOA and preferentially bind active RHOA proteins to block RHOA activity in neutrophils 10. These observations suggest that the function of RIPOR2 in cell migration is mediated mainly through RHOA inhibition.

In addition to its role in T cell migration, RIPOR2 is reported to act as a negative regulator of cell proliferation in a RHOA-independent manner. The RIPOR2 S5A mutant but not RIPOR2 RL151-152AA lost its antiproliferative effect 25. Previous studies showed that 14-3-3s are binding partners of HDAC6, a cytoplasmic deacetylase that promotes cancer cell proliferation in a deacetylase activity-dependent manner. HDAC6 regulated 14-3-3 interactions in a deacetylation activity-dependent manner 26. Furthermore, RIPOR2 inhibited the deacetylase activity of HDAC6. Consequently, phosphorylated RIPOR2 interacted with 14-3-3 and HDAC6 to form a tripartite complex 24-25, and this complex suppressed HDAC6 activity, resulting in disruption of the mitotic spindle formation and mitotic failure in resting T cells 25.

The active forkhead box O1 (FOXO1) transcription factor is required for maintaining the quiescence of T cells by promoting RIPOR2 transcription and expression 8,25. In T cells, T cell receptor (TCR) stimulation led to cytoplasmic localization of FOXO1 in a PI3K-AKT pathway-dependent manner 27. Notably, under TCR stimulation, RIPOR2 expression was reduced upon TCR activation 25. Thus, an increased cytoplasmic localization of FOXO1 upon TCR stimulation can be hypothesized to negatively regulate the expression of RIPOR2, which is essential for T cell proliferation.

In summary, these results indicate that RIPOR2 plays roles in the migration and proliferation of T cells by modulating the functions of RHOA and HDAC6, respectively.

### RIPOR2 and autophagy

Autophagy is a highly conserved process in all eukaryotes in which intracellular components are transported to lysosomes for degradation and recycling 28,29. Distinct from microautophagy and chaperone-mediated autophagy, macroautophagy is well established and involves the formation of autophagosomes 29. Autophagosomes carry and deliver cargo to lysosomes and then fuse with lysosomes to form autolysosomes, in which the contents are subsequently degraded 28-29. Autophagy functions as a major cytoprotective process by maintaining cellular homeostasis and recycling cytoplasmic contents, and dysfunction of autophagy has been associated with multiple human diseases 30-31.

Zhou et al. reported that RIPOR2 is involved in autophagy. A circRNA termed autophagy-related circular RNA (ACR) activates PINK1 expression by directly binding to DNMT3B and blocking DNMT3B-mediated promoter methylation of PINK1. RIPOR2 is a downstream target of PINK1. PINK1 phosphorylates RIPOR2 at serine 46, and phosphorylated RIPOR2 inhibits autophagy and cell death in cardiomyocytes 15. However, it remains unclear whether phosphorylated RIPOR2 governs autophagy in a RHOA-dependent manner.

### RIPOR2 and muscle cell differentiation

During the development of skeletal muscle, myoblasts from myotubes recognize other myoblasts, adhere, and fuse to form multinucleated myotubes; this process is critical in cellular differentiation and fusion 14,32. Multiple proteins, such as myogenic transcription factor (MRF4), neural cell adhesion molecule (N-CAM), and M-cadherin, are involved in these tightly regulated processes 13,33-34. Accumulating evidence suggests that RIPOR2 also functions as one of the fundamental regulators of myogenic differentiation 14,35.

In 2007, RIPOR2 was first characterized in human fetal muscle cell differentiation 14. RIPOR2 silencing reduced the protein level of myogenin, a marker of early myogenic differentiation, and abrogated primary human fetal muscle cell differentiation and fusion 14. RIPOR2 was also implicated in the formation of filopodia, which has been reported to be critical in key morphogenesis events at certain stages of muscle development 14,36. RIPOR2 binds to HDAC6 and dysferlin, the protein mutated in limb girdle muscular dystrophy 2B. The RIPOR2-HDAC6-dysferlin trimeric complex is transient, and RIPOR2 expression is necessary for the complex to form. Treatment of myogenic cells with pan-HDAC or HDAC6-specific inhibitors alters RIPOR2 expression. Inhibition of RIPOR2 expression in developing zebrafish results in abnormal muscle with low birefringence and myoseptal tears and in increased embryonic lethality 35.

RHOA has also been implicated in myoblast fusion 32. Active RHOA was found to induce lysosomal degradation of M-cadherin, one of the cell adhesion molecules involved in the fusion of myoblasts 33,37, and abrogated the interaction between M-cadherin and its partner p120-catenin, leading to inhibition of myoblast fusion 32. Given that RIPOR2 expression increases during muscle cell differentiation and that the amount of active RHOA decreases during myoblast fusion, it is possible that the effects of RIPOR2 on myoblast fusion events in membrane protuberances depend on RHOA inhibition. Therefore, RIPOR2 is likely to act as an inducible repressor of RHOA activity to promote cell-cell fusion during muscle or placenta formation.

N-CAM, another RIPOR2 binding partner, is also related to myoblast fusion 13,37-38. However, the functional impact of the N-CAM-RIPOR2 interaction on myoblast fusion is still unknown.

### RIPOR2 and hair cell functions

Cochlear hair cells are crucial for hearing due to their role in converting sound-induced vibration into electrical signals. This function relies on the specialized bundle of stereocilia at the apical surface of the hair cell 39. Stereocilia are tapered at their base near their site of insertion into the apical hair cell and are formed into a unique stairstep shape 40. Accumulating evidence suggests that the structure and dynamic properties of the actin core are essential for the functions of stereocilia 40.

Several studies have indicated that a loss-of-function mutation in RIPOR2 is correlated with hearing 41-44. Diaz-Horta et al. identified a splice site mutation (c.102-1G>A) in the RIPOR2 gene (MIM611410) that completely cosegregated with the phenotype in a large consanguineous Turkish family with recessive nonsyndromic, prelingual, profound hearing loss 41. The enrichment of RIPOR2 in the stereocilia of both inner and outer hair cells may suggest that a lack of functional RIPOR2 can hinder the development of the mechanotransduction apparatus. Moreover, knockdown of RIPOR2 causes hearing loss in zebrafish. A later report showed that RIPOR2 was localized in the basal taper of the mechanically sensitive stereocilia of hair cells, where oligomeric RIPOR2 interacted with RHOC to form the circumferential ring, an essential component of the taper region 42. RIPOR2 negatively regulates the structure and orientation of the hair cell stereociliary bundle via a mechanism based partially on the RIPOR2-nonmuscle myosin heavy chain 9 (MYH9) interaction to stabilize MYH9 43. MYH9-mediated coupling between microtubules and actomyosin is involved in the formation of nonmuscle myosin II (NMII) 45. Given that RIPOR2 inhibits HDAC6 activity and that HDCA6 destabilizes microtubule structures by promoting α-tubulin deacetylation, it might be possible that RIPOR2 contributes to microtubule flexibility rather than lengthening kinocilia in inner and outer hair cells 43,46.

However, it might be possible that RIPOR2 functions in maintaining normal hearing through mechanisms other than affecting the MYH9 abundance or inhibiting HDAC6 activity 44. An in-frame deletion (c.1696_1707del) mutation in RIPOR2 has been linked with hearing loss 44. Strikingly, this mutation did not alter RIPOR2's function in filopodia formation, suggesting that a RHO-independent mechanism may exist. Further studies are needed to clarify the RHO-dependent and RHO-independent mechanisms of RIPOR2 in mediating hair cell function.

### RIPOR2 and cancer

According to data from The Cancer Genome Atlas 47, the expression of RIPOR2 is downregulated compared to that in normal tissues in the majority of cancer types, especially in uterine corpus endometrial carcinoma and diffuse large B-cell lymphoma. However, the precise roles of RIPOR2 in tumorigenesis and progression are still poorly understood.

Zhang K et al. reported that RIPOR2 was upregulated in PC3 holoclones with cancer stem cell characteristics. PC3 cells, a line of androgen-independent human prostate cancer cells, were divided into three morphologically distinct colonies: holoclones, meroclones, and paraclones. Compared with meroclones and paraclones, holoclones exhibited increased self-renewal activity and proliferation capability. In addition, gene expression patterns differed among the three colony types. The expression of RIPOR2 was significantly upregulated in PC3 holoclones. Furthermore, PC3 prostate tumor-initiating cells with the molecular profile RIPOR2^high^/melanotransferrin (MFI2)^low^/LEF1^low^ increased tumor angiogenesis 48. Despite these findings, the molecular mechanisms underlying RIPOR2-related angiogenesis are still unknown.

### The pathophysiological functions of RIPOR1 and RIPOR3

**Figure 3:**
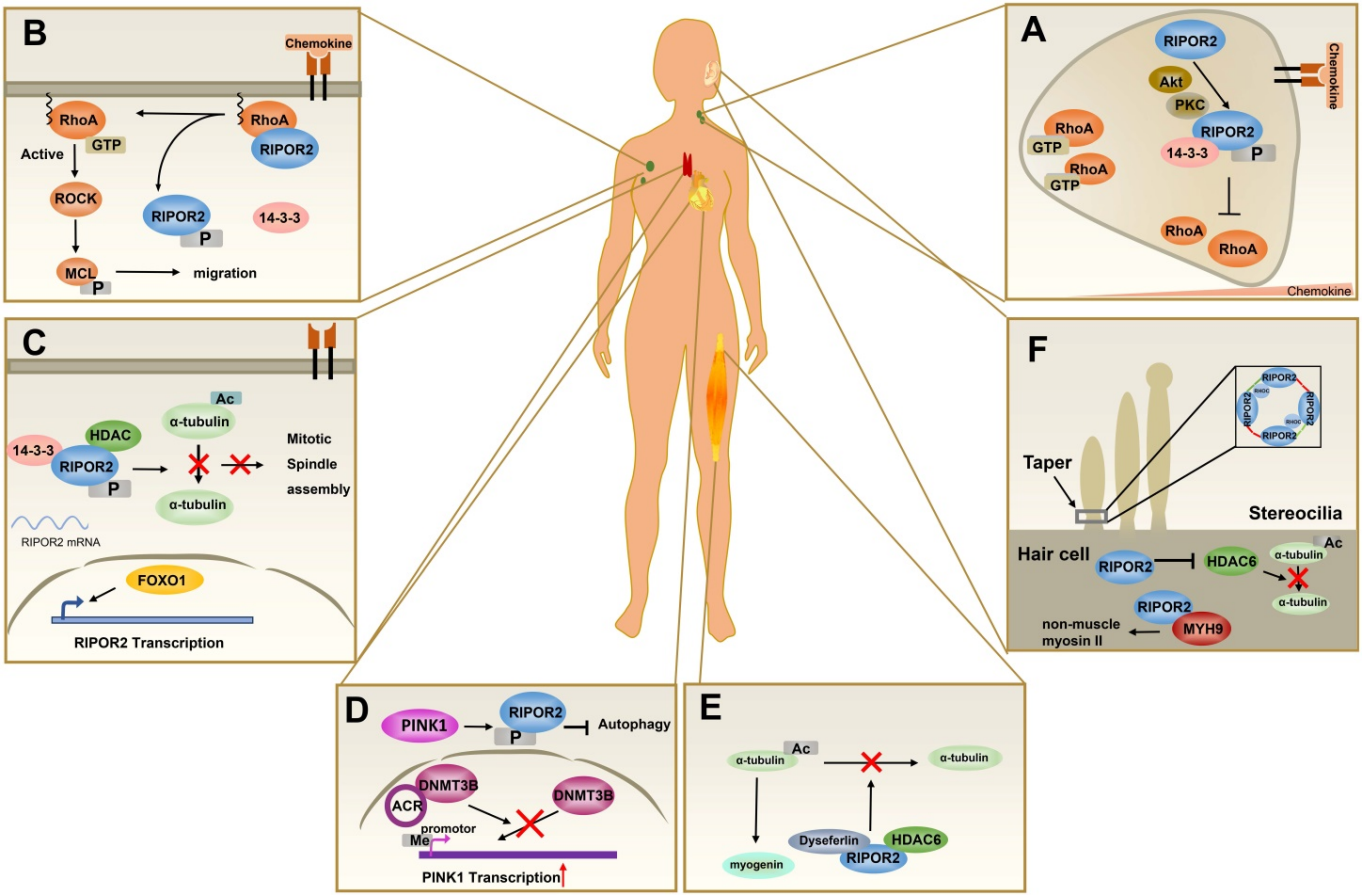
** The pathophysiological functions of RIPOR2. (A)** In primary neutrophils, the front localization of phosphorylated RIPOR2 stabilized by 14-3-3 leads to unequal distribution of active RHOA. **(B)** In resting T cells, RIPOR2 combines with RHOA and exerts suppressive effects on its activity at the plasma membrane to maintain immune cells in a quiescent state. Upon chemokine stimulation, 14-3-3 interacts with phosphorylated RIPOR2 by protein kinases, breaking the RIPOR2-RHOA interaction and relieving RHOA inhibition. **(C)** In resting T cells, FOXO1 upregulates RIPOR2 protein level, and then phosphorylated RIPOR2 binds to 14-3-3 and HDAC6 to form tricomplex, inhibiting deacetylation of α-tubulin and thereby discouraging mitotic spindle assembly. **(D)** By interacting with DNMT3B, ACR suppresses DNA methylation of PINK1 to upregulate the expression of PINK1, increasing RIPOR2 phosphorylation, which contributes to autophagy inhibition and cell death in cardiomyocytes. **(E)** Tricomplex formed by HDAC6, RIPOR2, and dysferlin inhibits the deacetylation activity of HDAC6, resulting in the accumulation of acetylated α-tubulin, which is believed to promote muscle cell differentiation. **(F)** RIPOR2 is located in the basal taper of mechanically sensitive stereocilia of hair cells, where it interacts with RhoC to form a circumferential ring--RIPOR2 oligomerization, a structure necessary for the taper region. As well as stabilizing MYH9 in hair cells, RIPOR2 inhibits HDAC6 activity in order to regulate stereociliary bundle structure.

Among the RIPOR family members, RIPOR2 is the best studied. As described above, RIPOR2 is involved in a plethora of physiological and pathological processes, such as immune cell polarization, migration, and autophagy (Figure 3). Despite their structural similarities, RIPOR1 and RIPOR3 show potentially distinct functional features relative to RIPOR2.

**Figure 4:**
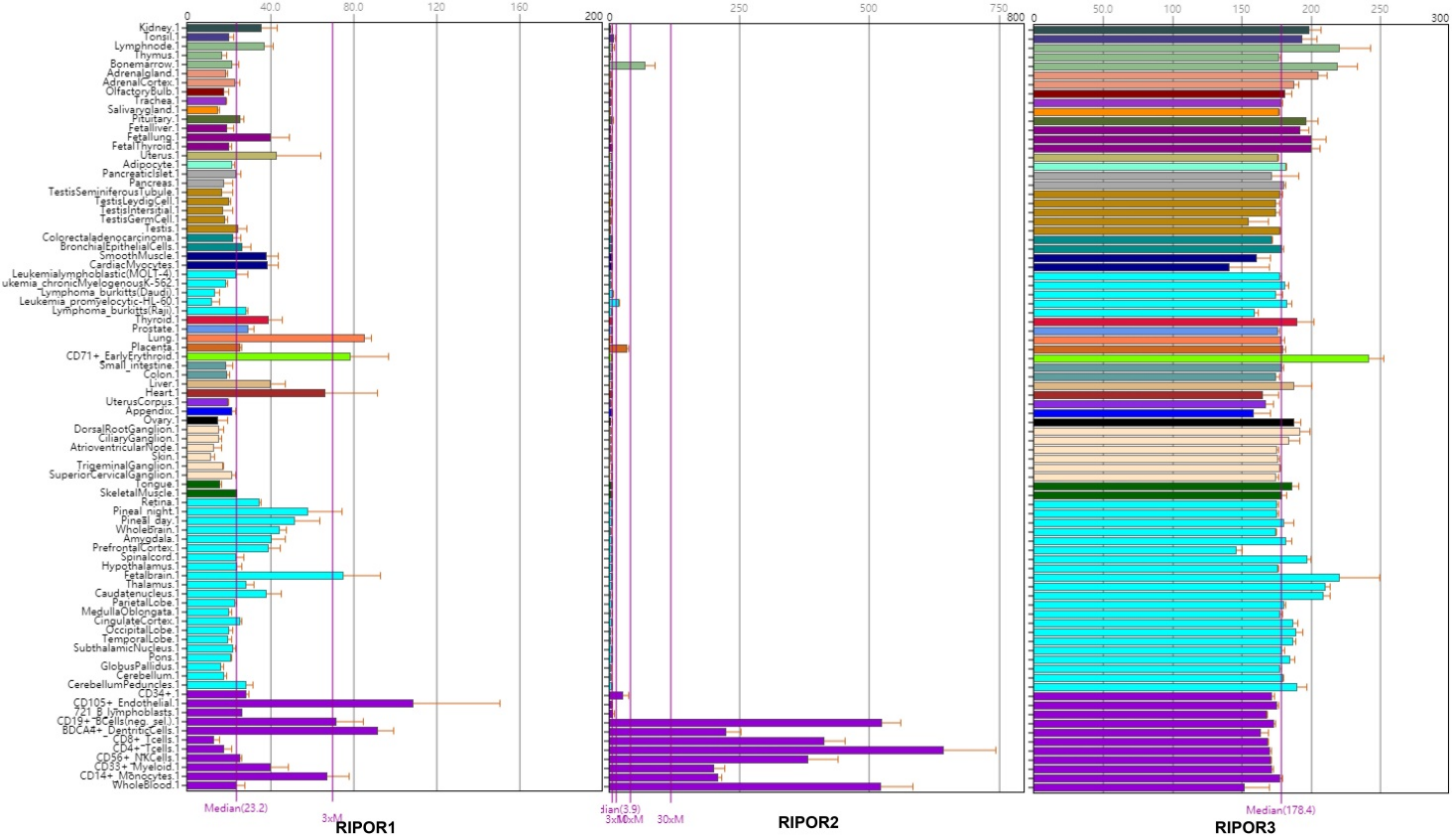
High-density oligonucleotide arrays are used to examine patterns of gene expression in a panel of 79 human tissues. The data is from BioGPS.

To better understand the potential functions of the RIPOR proteins, we examined the mRNA expression patterns of RIPORs in 79 human tissues 49 (Figure 4). RIPORs are differentially expressed in human tissues. RIPOR2 is predominantly expressed in immune cells, including T cells, B cells, NK cells, monocytes and myeloid cells. Our previous study showed that the RIPOR2 mRNA level is much higher in bone marrow neutrophils than in other cell types 10. In contrast, RIPOR1 and RIPOR3 are ubiquitously expressed in the majority of tissues. The distinct expression patterns of RIPORs suggest the different biological functions performed by these proteins. RIPOR1 regulates Golgi apparatus reorientation during HeLa cell migration. It functions as a scaffold protein that links RHO proteins to Golgi-localizing proteins (Cerebral Cavernous Malformation-3 (CCM3) and mammalian STE20-like protein kinase 3 (MST3), and MST4), resulting in relocalization of Golgi-localized proteins from the Golgi apparatus toward the leading edge of cells during directional cell migration. In response to wounding, the Golgi apparatus was found to be reoriented in HeLa cells at the edge of the wound. Depletion of RIPOR1 impeded Golgi apparatus reorientation and directional migration of HeLa cells following wounding. This effect was consistent with that of RHOA activity inhibition 11. Therefore, the divergent interacting proteins of RIPORs may account for their distinct functions (Table 1).

Analysis of RNA-seq data in The Cancer Genome Atlas showed that RIPOR1 is upregulated in gastrointestinal tumors such as cholangiocarcinoma, colon adenocarcinoma, esophageal carcinoma, and liver hepatocellular carcinoma. Intriguingly, the expression level of RHOA in these gastrointestinal tumors is also increased 47. Given that RHOA is generally considered an oncogene 50, we hypothesize that RIPOR1 may act as an oncogene or tumor suppressor in a context-dependent manner, although further investigations are warranted in the future.

Likewise, little is known about the biological functions of RIPOR3 in cells. As no studies have yet examined RIPOR3, we can only speculate from its mRNA expression profile that the functions of RIPOR3 may differ from those of RIPOR1 and RIPOR2. Further investigations are needed to decipher the biological functions of RIPOR3 and determine whether these functions are altered in pathological conditions.

## Conclusions

These findings illustrate the unity and versatility of the RIPOR family in health and disease. As a first insight, the RIPORs share many similarities in amino acid sequences, protein interaction partners, and biological functions. Moreover, the differences in their roughly homologous amino acid sequences might account for their varying functions through interactions with proteins in addition to RHO (A, B, and C).

Here, we highlight the roles of RIPORs in physiological and pathological contexts - especially roles linked with the actin cytoskeleton, including cell polarization, migration, adhesion, and organelle trafficking - based on studies conducted in this decade. Currently, it is clear that the regulation of RHO GTPase functions is shared by three members, but their other molecular functions are still poorly understood. Given their sequence divergence and tissue-specific expression, RIPORs are expected to function in diverse biological processes. More research is needed to clearly clarify the roles of RIPORs in homeostasis and disease.

## Figures and Tables

**Table 1:** Interactors of RIPOR Family

Protein	Interactors	Process/Pathway Affected	Cells	Reference
RIPOR1	RHOA	linking active RHO subfamily and Golgi-localizing proteins to regulate Golgi reorientation	HeLa cells	11
RIPOR2	RHOA	Inhibiting RHOA activity to negatively regulate immune cell migration	T lymphocytes Primary neutrophils	8-10
	HDAC6, 14-3-3	Interacting with HDAC6 deacetylase and 14-3-3 to inhibit T-cells proliferation	T lymphocytes	25
	HDAC6, Dysferlin	Interacting with HDAC6 and dysferlin to promote myogenic differentiation	Primary muscle cells	35
	HDAC6	Inhibiting HDAC6 activity to maintain the structure and function of the auditory hair cell bundle	Hair cells	43
	MYH9	Stabilizing non-muscular myosin heavy chain 9(MYH9) to maintain the structure and function of the auditory hair cell bundle	Hair cells	43
	RHOC	Interacting with RHOC to form a RIPOR2 ring-like structure critical for mechanosensory hair cell function	Hair cells	42
	N-CAM	Interacting with NCAM	-	13
RIPOR3	unknown	-	-	-

## Abbreviations

RIPOR: the RHO family interacting cell polarization regulators
GEFs: guanine nucleotide exchange factors
GAPs: GTPase activating proteins
GDIs: guanine nucleotide dissociation inhibitors
MST4: mammalian STE20-like protein kinases 4
PKN1: protein kinase N-1
RTKN1: Rhotekin
RHPN1: Rhophilin
PLC: Phospholipase C
PI3K: phosphatidyl-inositol-3-kinase
PINK1: phosphatase and tensin homolog-induced putative kinase 1
HDAC6: histone deacetylase 6
FOXO1: forkhead box O1
TCR: T cell receptor
DNMT3B: DNA methyltransferase 3 beta
MRF4: myogenic transcription factor
N-CAM: neural cell adhesion molecule
MYH9: non-muscular myosin heavy chain 9
NMII: non-muscle myosin II
MFI2: melanotransferrin
CCM3: Cerebral Cav-ernous Malformation-3 protein
MST3: Mammalian STE20-like protein kinases 3
